# Subacute to chronic Alzheimer-like alterations after controlled cortical impact in human tau transgenic mice

**DOI:** 10.1038/s41598-019-40678-4

**Published:** 2019-03-07

**Authors:** Yanchong Zhang, Feng Wu, Khalid Iqbal, Cheng-Xin Gong, Wen Hu, Fei Liu

**Affiliations:** 1 Department of Neurochemistry, Inge Grundke-Iqbal Research Floor, New York State Institute for Basic Research in Developmental Disabilities, Staten Island, New York USA; 2 Key Laboratory for Neuroregeneration of Ministry of Education and Co-innovation Center for Neuroregeneration of Jiangsu Province, Nantong University, Nantong, Jiangsu China

## Abstract

Repetitive traumatic brain injury (TBI) has been linked to late life development of chronic traumatic encephalopathy (CTE), a neurodegenerative disorder histopathologically characterized by perivascular tangles of hyperphosphorylated tau at the depth of sulci to later widespread neurofibrillary pathology. Although tau hyperphosphorylation and neurofibrillary-like pathology have been observed in the brain of transgenic mice overexpressing human tau with aggregation-prone mutation after TBI, they have not been consistently recapitulated in rodents expressing wild-type tau only. Here, we characterized Alzheimer-like alterations behaviorally, biochemically and immunohistochemically 6 weeks and 7 months after unilateral mild-to-moderate controlled cortical impact (CCI) in 5–7-month-old Tg/htau mice, which express all six isoforms of non-mutated human tau in a mouse tau null background. We detected hyperphosphorylation of tau at multiple sites in ipsilateral hippocampus 6 weeks but not 7 months after CCI. However, neuronal accumulation of AT8 positive phospho-tau was sustained in the chronic phase, in parallel to prolonged astrogliosis, and decreased neural and synaptic markers. The mice with CCI also exhibited cognitive and locomotor impairment. These results indicate subacute to chronic Alzheimer-like alterations after CCI in Tg/htau mice. This is the first known study providing insight into the role of CCI in Alzheimer-like brain alterations in young adult mice expressing only non-mutated human tau.

## Introduction

Head trauma is the leading cause of death and disability in children and adults from 1 to 44 years of age; traumatic brain injury (TBI) represents the major type of trauma which leads to morbidity, disability and mortality^1^. At least 5.3 million Americans, or ~2% of the U.S. population, currently live with disabilities resulting from TBI^2,3^. American football players, boxers and soldiers deployed in war are at high risk of exposure to TBI, particularly repetitive mild TBI^4–7^. Of the long-term effects of TBI on survivors, cognitive impairment is widely accepted as the most devastating deficit which precludes them from being re-integrated into the society^8^.

Epidemiological studies have suggested TBI as a major risk factor for late life development of cognitive impairment and Alzheimer’s disease (AD)^9–13^. Chronic traumatic encephalopathy (CTE) has been known for many years to be present in many individuals exposed to repetitive, often mild or concussive head injury as in boxers and American football players^14–19^; whereas a recent study has shown widespread tau and amyloid-β pathologies, the two hallmarks of AD, many years after a single TBI in humans^20^.

AD is multi-factorial and involves several different etiopathogenic mechanisms^21,22^. The familial form of AD, which accounts for less than 1% of all cases, is caused by certain point mutations in β-amyloid precursor protein (APP), presenilin 1 or presenilin 2 genes^23,24^. The exact causes of sporadic forms of AD, which account for over 99% of the cases, are not yet understood. Histopathologically, the familial and the sporadic forms of AD are indistinguishable from each other; they are both characterized by neurodegeneration of the brain, especially the hippocampus and the neocortex that are associated with numerous intraneuronal neurofibrillary tangles (NFTs) and extracellular deposits of β-amyloid as cores of neuritic/senile plaques. Clinicopathological correlation studies have reported that the density of NFTs but not of Aβ plaques correlates with the degree of dementia in AD patients^25–27^. In addition to AD, neurofibrillary pathology, which comprises abnormally hyperphosphorylated tau^28,29^, is also a hallmark of a family of related neurodegenerative diseases called tauopathies, which include, but not limited to, dementia pugilistica or CTE, frontotemporal dementia-tau, corticobasal degeneration, Pick disease, and Guam Parkinsonism dementia complex^30,31^.

The causal role of repetitive TBI in the development of CTE is well recognized in clinical settings^32,33^. Although tau hyperphosphorylation and tau pathology after TBI have been observed using transgenic mice overexpressing human tau with aggregation-prone mutation^34^, they have not been consistently and assuredly recapitulated in rodents expressing wild-type tau only^35–38^. It remains to be characterized whether and to what extent tau hyperphosphorylation and filamentous tau pathology can be mimicked in young adult mice which express relatively physiological level of non-mutated human tau.

To study the role of TBI in AD-like alterations in an animal model that may mimic pathophysiological changes of tau and associated proteins in the human brain after TBI more closely, we performed controlled cortical impact (CCI) in human tau transgenic mice, in which all six isoforms of non-mutated human tau are expressed in a mouse tau null background^39^ and analyzed AD-like alterations behaviorally, biochemically and immunohistochemically. We found hyperphosphorylation of tau and sustained accumulation of phospho-tau in parallel to astrogliosis, decreased neural and synaptic markers, and cognitive and locomotor impairment after CCI.

## Results

### CCI causes learning and memory deficits and motor coordination impairment

**Figure 1:**
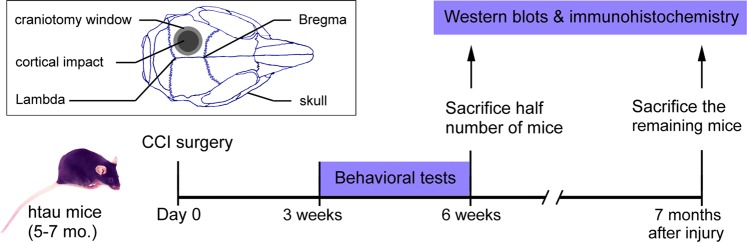
Study design. Tg/htau mice, 5–7 months of age, were subjected to controlled cortical impact (CCI) of the left parietal cortex. Neurobehavioral tasks, which included open field, elevated plus maze, novel object recognition, rota-rod and Morris water maze tests, were performed between 3 and 6 weeks after injury. One half number of mice were euthanized at 6 weeks and the remaining mice at 7 months after CCI for the assessment of subacute and chronic biochemical/immunohistological alterations of the brain, respectively. The insert is a schematic diagram showing the site of craniotomy and cortical impact.

**Figure 2:**
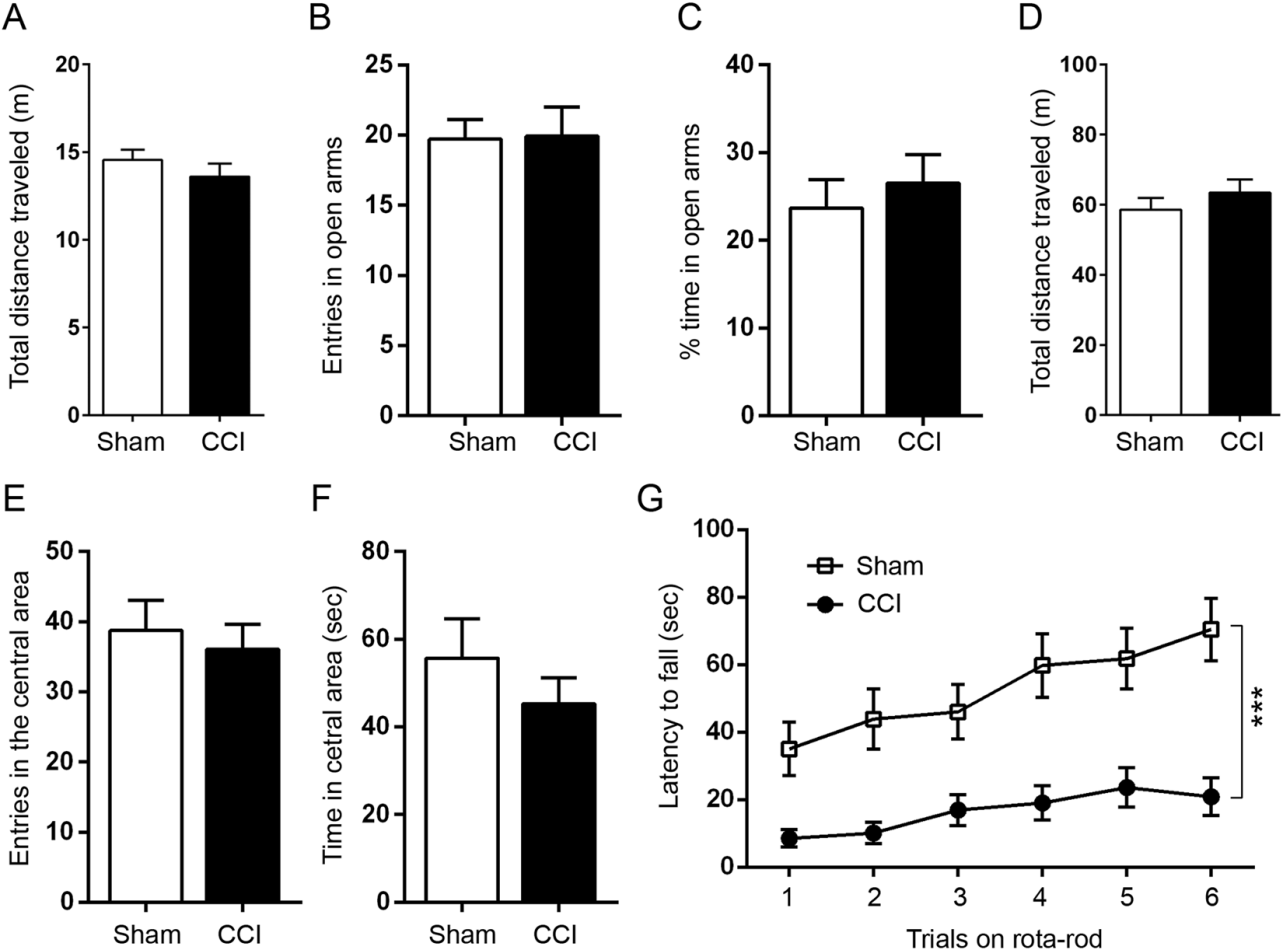
CCI leads to impairment in motor coordination but not in spontaneous locomotor activity or anxiety in Tg/htau mice. (**A**–**C**) Elevated plus maze to test anxiety. (**D**–**F**) Open field test to assess spontaneous activity and anxiety-like behavior. (**G**) Accelerating rota-rod test to examine motor strength/coordination. Mice with CCI showed significant decrease in latency to fall off the rotating rod, as compared to sham control mice. Data are expressed as mean ±SD (n = 13–15 mice/group) and analyzed by unpaired Student *t* test (**A**–**F**) or repeated measures ANOVA followed by Bonferroni’s *post hoc* test (**G**). ***P < 0.001.

To learn the effect of TBI on motor coordination and learning and memory, we introduced unilateral CCI in the left parietal cortex in Tg/htau mice at 5–7 months of age, and performed a battery of behavioral tests between 3 and 6 weeks post CCI (Fig. 1). The mice were first subjected to elevated plus maze test, an established task for the assessment of anxiety^40^. We found that CCI and sham mice traveled comparable total distance on the elevated plus maze (Fig. 2A), and exhibited similar probability to enter the open arms, with no statistical difference in the number of entries and total time spent in the open arms between the two groups (Fig. 2B,C). The mice were also subjected to open field test for exploratory activity, anxiety and spontaneous locomotor activity. We found that CCI and sham mice traveled a similar total distance, showed similar entries into and similar time spent in the central area (Fig. 2D–F). These data suggest that CCI did not significantly alter exploratory activity, anxiety or spontaneous locomotor activity in Tg/htau mice. However, when motor coordination was examined by rota-rod test^41^, we found a marked decrease in latency to fall in CCI mice compared to sham control animals (Fig. 2G), suggesting that motor strength/coordination was impaired in mice with CCI.

**Figure 3:**
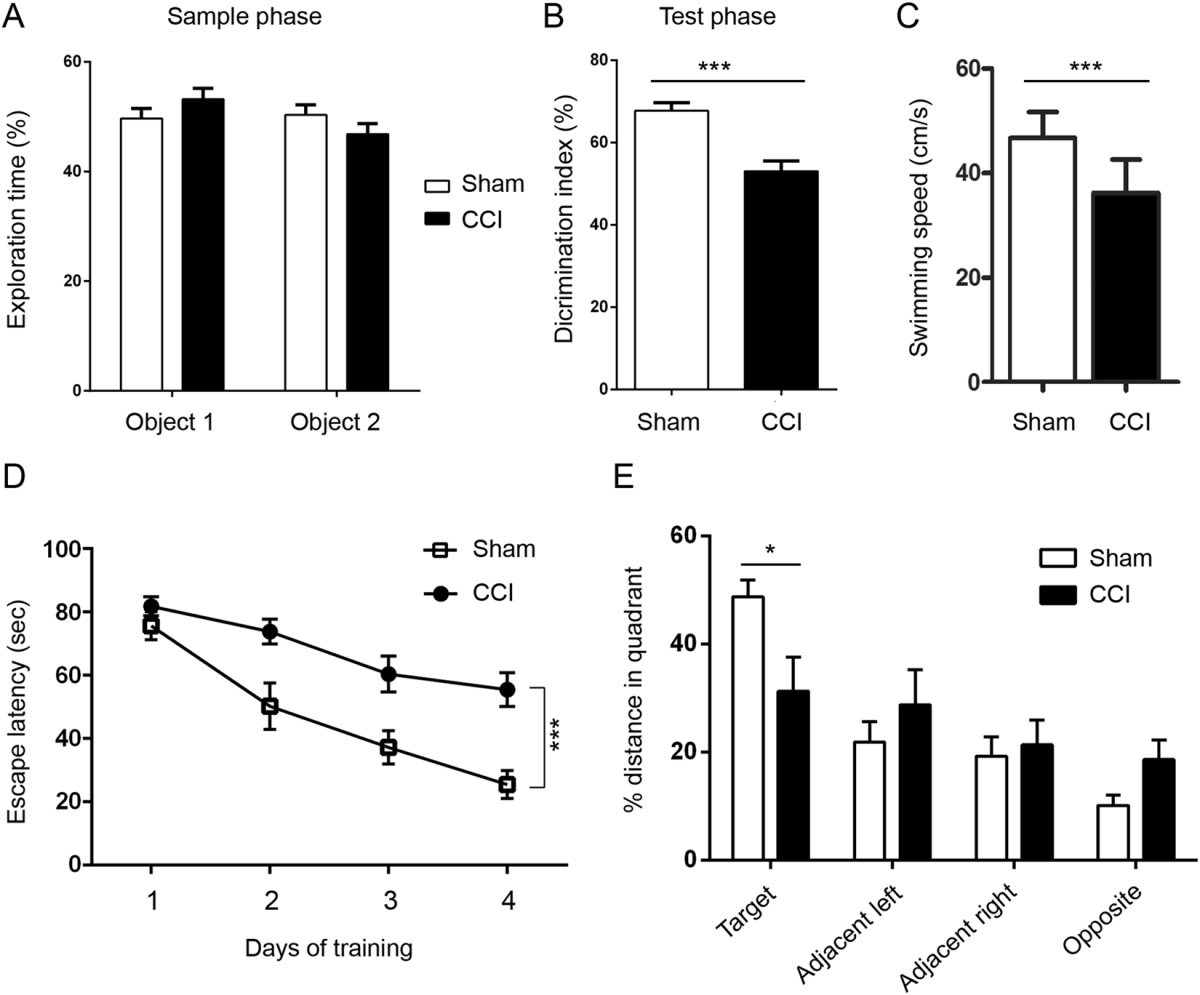
CCI impairs learning and memory in Tg/htau mice. (**A**,**B**) Novel object recognition to evaluate episodic memory. (**C**–**E**) Morris water maze to test learning and spatial reference memory. Mice after CCI showed significant impairment in episodic and spatial reference memories and reduced swimming speed, as compared to sham control mice. Data are expressed as mean ±SD (n = 13–15 mice/group) and analyzed by matched observation ANOVA followed by Bonferroni’s *post hoc* test (**A**), unpaired Student *t* test (**B**,**C**) or repeated measures ANOVA followed by Bonferroni’s *post hoc* test (**D**,**E**). *P < 0.05, ***P < 0.001.

We employed novel object recognition to test episodic memory in mice. We found that CCI mice showed markedly reduced discrimination between novel and familiar objects as compared to the sham control animals (Fig. 3A,B). In Morris water maze task, the CCI mice showed reduced swim speed (Fig. 3C), impaired acquisition of spatial reference memory (Fig. 3D) and a decreased capability of locating the target in the probe trial 24 h after the last training session (Fig. 3E). These data suggest that both episodic and spatial reference memories were impaired in Tg/htau mice after CCI.

### CCI induces accumulation of hyperphosphorylated tau in the brain

**Figure 4:**
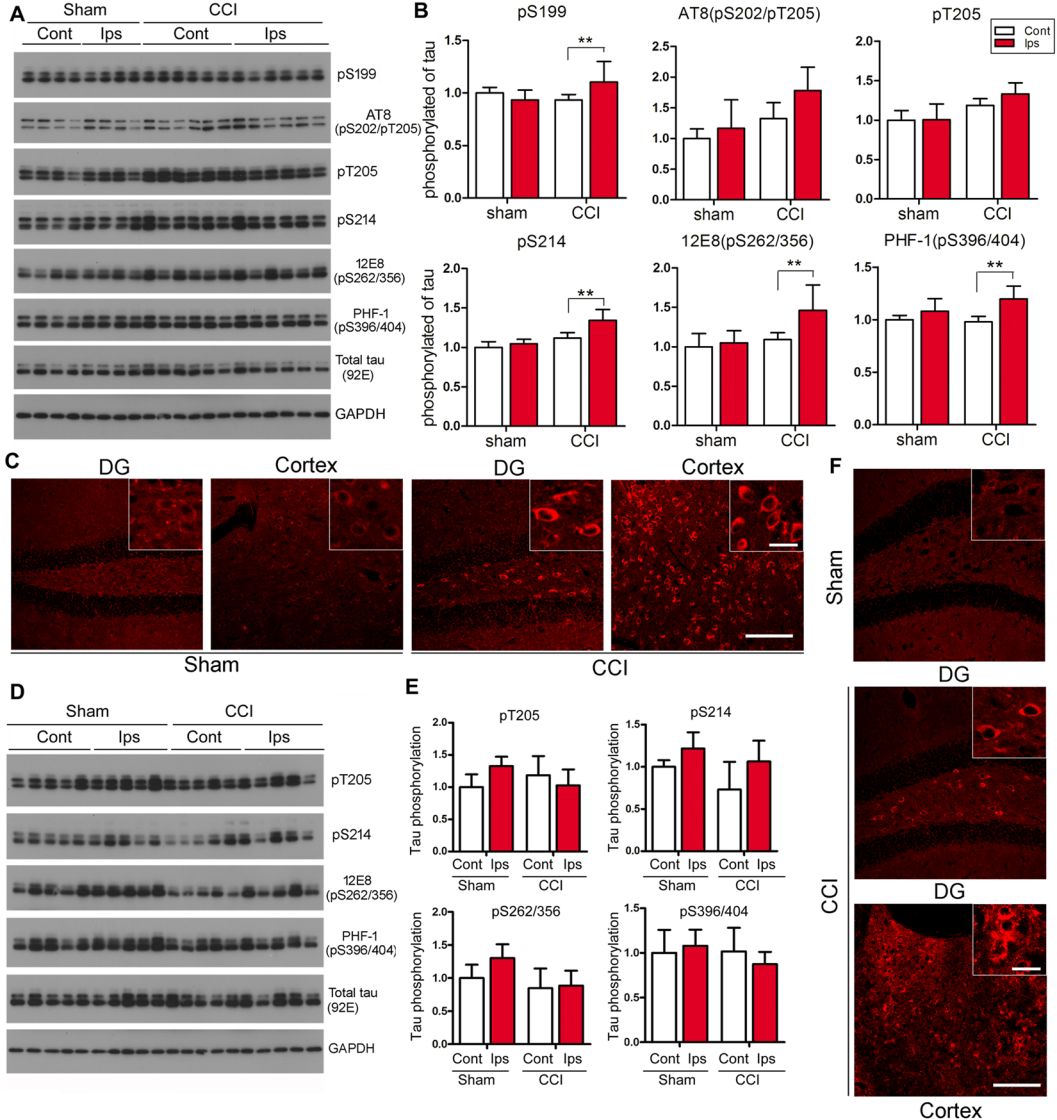
CCI induces accumulation of hyperphosphorylated tau in the brain of Tg/htau mice. (**A**) Western blots and (**B**) quantification showing the level of tau phosphorylated at the indicated sites in the hippocampus 6 weeks after CCI. (**C**) Representative photomicrographs showing AT8 staining in the ipsilateral dentate gyrus (DG) and cerebral cortex 6 weeks after CCI. Numerous AT8-positive neuronal profiles were seen in the hilus and the ipsilateral cortex. (**D**) Western blots and (**E**) quantification showing the level of phosphorylated tau at indicated sites in the hippocampus 7 months after CCI. (**F**) Representative photomicrographs showing AT8 staining in DG and cerebral cortex 7 m after CCI. Data are expressed as mean ±SD (n = 5–7 mice/group) and analyzed by matched observation ANOVA followed by Bonferroni’s *post hoc* test. **P < 0.01 for phosphorylation sites in which interaction showed significant difference. Inserts show high magnification views correspondingly. Bar = 100 μm for all low magnification views and 20 μm for all inserts. *Ips* ipsilateral, *Cont* contralateral.

To study tau phosphorylation in the hippocampus after CCI, we analyzed the level of phosphorylated tau by Western blots developed with site-specific and phosphorylation-dependent anti-tau antibodies 6 weeks post CCI. We found that levels of tau phosphorylated at Ser^199^, Ser^214^, Ser^262/356^ and Ser^396/404^, when normalized with total tau, were slightly increased in the ipsilateral hippocampi of mice 6 weeks after CCI (Fig. 4A,B). The level of tau phosphorylated at Ser^202^/Thr^205^ by antibody AT8 and at Thr^205^ by a polyclonal antibody also showed a trend of increase in ipsilateral hippocampus which did not reach statistical significance (Fig. 4A,B). These data suggest subacute hyperphosphorylation of tau in the hippocampus post-CCI. To learn whether CCI induces tau pathology on a subacute basis, we immunostained brain sections 6 weeks post CCI with AT8, an phospho-tau antibody which reveals pre-tangle stage of neurofibrillary pathology^42^. We found robust somatodendritic AT8 staining in dentate gyrus of the hippocampus and in cerebral cortex (Fig. 4C); there was no apparent AT8 staining in corresponding brain regions in sham control mice (Fig. 4C). These results suggest that CCI can induce accumulation of hyperphosphorylated tau in Tg/htau mice on a subacute basis.

To study how hyperphosphorylation of tau evolves on a chronic basis after CCI, we determined tau hyperphosphorylation and tau pathology with Western blots and immunohistochemistry 7 months after CCI. Surprisingly, we found no statistically significant difference in the level of tau phosphorylated at Thr^205^, Ser^214^, Ser^262/356^, and Ser^396/404^ between the ipsilateral and contralateral hippocampi (Fig. 4D,E) or between the CCI and sham groups. However, robust AT8 immunoreactivity was still observed in the somatodendritic compartment of neurons in the dentate gyrus and cerebral cortex (Fig. 4F), suggesting long-lasting tau pathology induced by CCI.

### CCI does not affect alternative splicing of tau

**Figure 5:**
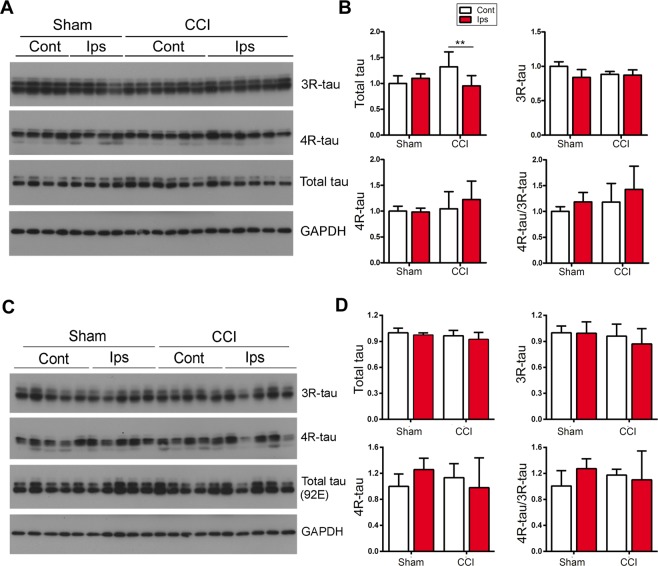
Alternative splicing of tau exon 10 is not dysregulated in CCI mouse brain on a subacute or chronic basis. (**A**) Western blots and (**B**) quantification showing the levels of 3R-tau, 4 R tau and total tau in the hippocampus 6 weeks after CCI. (**C**) Western blots and (**D**) quantification data showing the levels of 3R-tau, 4 R tau and total tau in the hippocampus 7 months after CCI. Data are expressed as mean ± SD (n = 5–7 mice/group) and analyzed by matched observation ANOVA followed by Bonferroni’s *post hoc* test. **P < 0.01. *Ips* ipsilateral, *Cont* contralateral.

Alternative splicing of tau exon 10, which leads to generation of either 3R-tau or 4R-tau, has been implicated in tau pathogenesis^43^. Due to expression of normal human tau isoforms in a murine tau null background^39^, the Tg/htau mouse model provides a unique opportunity to study the role of tau alternative splicing in tau pathogenesis in TBI. To determine the effect of CCI on alternative splicing of tau exon 10, we analyzed levels of 3R-tau and 4R-tau in ipsilateral and contralateral hippocampi by Western blots 6 weeks post CCI. We found that levels of 3R-tau and 4R-tau were similar in both ipsilateral and contralateral hippocampi of CCI and sham mice (Fig. 5A,B). However, the level of total tau was slightly decreased in the ipsilateral than the contralateral hippocampus in mice with CCI (Fig. 5A,B). We further studied the chronic effect of CCI on tau exon 10 splicing, by determining levels of 3R-tau, 4R-tau and total tau in the hippocampi 7 months after CCI. However, we found no significant difference between sham and CCI hippocampi in levels of 3R-tau, 4R-tau or total tau (Fig. 5C,D). Taken together, these data suggest that CCI may not affect tau exon 10 splicing on a sub-acute to chronic basis.

### CCI induces astrogliosis

**Figure 6:**
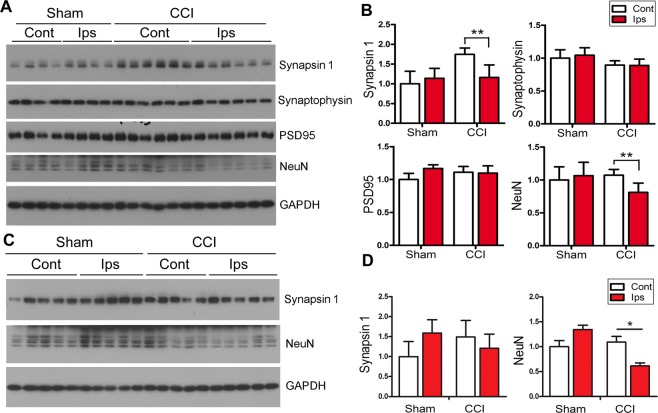
The hippocampus shows decreased neuronal/synaptic markers after CCI in Tg/htau mice. (**A**) Western blots and (**B**) quantification showing levels of synapsin 1, synaptophysin, PSD-95 and NeuN in the hippocampus 6 weeks after CCI. (**C**) Western blots and (**D**) quantification data showing the levels of synapsin 1 and NeuN in the hippocampus 7 m after CCI. Data are expressed as mean ± SD (n = 5–7 mice/group) and analyzed by matched observation ANOVA followed by Bonferroni’s *post hoc* test. *P < 0.05, **P < 0.01. *Ips* ipsilateral, *Cont* contralateral.

To learn the effect of CCI on synaptic plasticity and neuronal survival, we analyzed levels of pre-synaptic (synapsin-1 and synaptophysin), post-synaptic (PSD-95) and neuronal (NeuN) markers in the hippocampi of Tg/htau mice 6 weeks post-CCI. We found that the level of synapsin-1 was decreased in ipsilateral compared to contralateral hippocampus (Fig. 6A). However, levels of PSD-95 and synaptophysin showed no significant change after CCI (Fig. 6A,B). Reduced level of NeuN was observed in the ipsilateral as compared to contralateral hippocampus (Fig. 6A). Interestingly, hippocampal synapsin-1 level did not show significant decrease 7 months after CCI, whereas the level of NeuN remained decreased in ipsilateral than in contralateral hippocampus (Fig. 6C,D).

**Figure 7:**
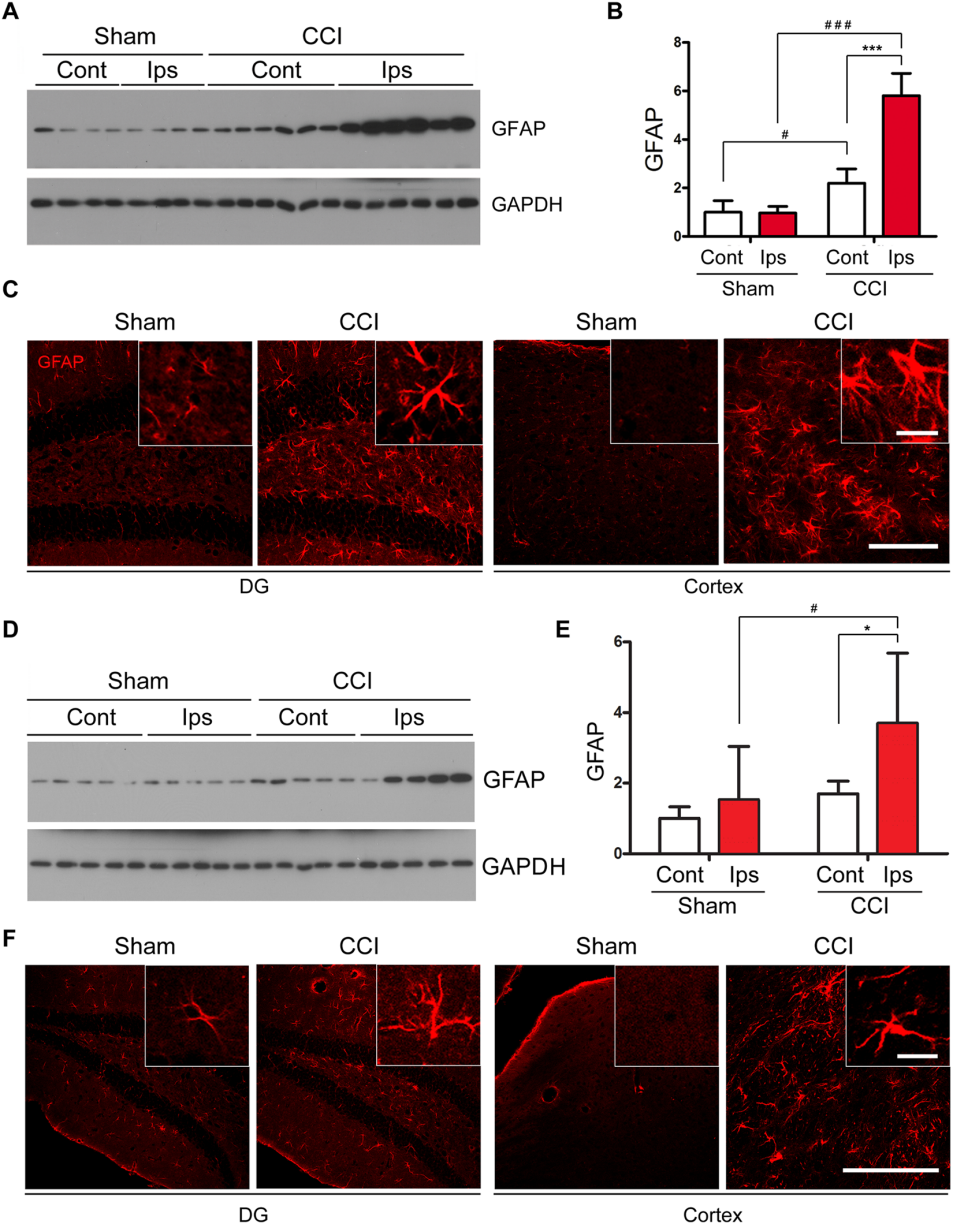
CCI induces astrogliosis in the brain of Tg/htau mice. (**A**) Western blots and (**B**) quantification showing the level of GFAP in the hippocampus 6 w after CCI. (**C**) Immunostaining of mouse brain sections for GFAP 6 w after CCI. (**D**) Western blots and (E) quantification showing GFAP level in the hippocampus 7 m after CCI. (**F**) Immunostaining of mouse brain for GFAP 7 m after CCI. Data are expressed as mean ± SD (n = 5–7 mice/group) and analyzed by matched observation ANOVA followed by Bonferroni’s *post hoc* test. *P < 0.05, ***P < 0.001, ipsilateral v.s. contralateral; ^#^*P* < 0.05, ^###^*P* < 0.001 v.s. sham. Inserts show high magnification views correspondingly. Bar = 100 μm for all low magnification views and 20 μm for all inserts. *Ips* ipsilateral, *Cont* contralateral.

Gliosis is a common feature of neurodegenerative disorders including CTE and plays a critical role in pathogenesis in these diseases; reactive gliosis is commonly seen in animal models of TBI^44^. To evaluate astrogliosis in the CCI mouse brains, we analyzed GFAP, a marker for astroglia, by Western blots in the mouse hippocampi 6 weeks after CCI. We found that the level of GFAP was markedly increased in the ipsilateral hippocampus as compared to the contralateral and sham controls (Fig. 7A,B). Immunohistochemical staining of brain sections for GFAP showed markedly increased number of astrocytes in the dentate gyrus and cerebral cortex (Fig. 7C). These results suggest subacute astrogliosis in the brain after CCI.

To evaluate CCI-induced chronic astrogliosis, we analyzed GFAP expression in the hippocampus 7 months post CCI. We found that the level of GFAP was higher in ipsilateral hippocampus than that in the sham control (Fig. 7D,E). GFAP immunostaining confirmed chronic astrogliosis in cortex and the hippocampus 7 m after CCI (Fig. 7F).

## Discussion

CCI is a popular animal model of TBI owing to desirable control over injury parameters, reproducibility and high survivability^45,46^. In the present study, CCI of the parietal cortex in Tg/htau mice produced impairment in episodic and spatial reference memories and motor strength/coordination, as detected by novel object recognition, Morris water maze and rota-rod test, but not in general locomotor activity determined by open-field test. This is in line with a previous study which reported that CCI in the parietal cortex leads to impairment in cognition and torso flexion but not general locomotion in adult C57BL/6 mice^47^. Importantly, we observed a ~20% decrease in swimming speed in Morris water maze and substantially reduced latency to fall from rota-rod in mice with CCI as compared to sham control, suggesting that 1.5-mm-deep deformation of the parietal cortex tissue damages brain regions responsible for locomotor function, at least motor strength/coordination. This could have been a factor that might confound certain readouts of cognitive assessment, for instance escape latency in Morris water maze task. However, impairment in learning and memory is evidenced by other indices relatively independent of motor function, including percent distance travelled in target quadrant in Morris water maze and discrimination index in novel object recognition. In the present study, we observed no significant change in anxiety-like behavior as detected by elevated plus maze and open field tests; this is discrepant with the findings of a previous study^47^, possibly due to different strains of mice used. The discrepancy between decreased swimming speed and similar total distance traveled in open field could be attributed to different stringency of the tasks.

In the present study, we sought out to investigate whether and to what extent hyperphosphorylation and change in alternative splicing of tau occur after CCI in Tg/htau mice. This line of mice was utilized in that it expresses all six isoforms of non-mutated human tau in a murine tau null background^39^. Murine tau of wild type mice is less prone to hyperphosphorylation and aggregation than human tau^35^. Western blot data revealed that the human tau protein was hyperphosphorylated at multiple sites, including Ser^199^, Ser^214^, Ser^262/356^ and Ser^396/404^, in the brain of Tg/htau mice 6 weeks after CCI. Hyperphosphorylation of tau is likely the consequence of imbalanced tau kinase/phosphatase activities resulted from direct neural tissue injury and secondary damages that follow^45^. CCI is known to trigger activation of calpain^48,49^, which in turn cleaves and activates kinases or kinase regulators that are involved in phosphorylation of tau, including P35 (via cdk5)^50^, Dyrk1A^51^ and GSK-3β^52^. CCI may also lead to tau hyperphosphorylation through other pathways, for instance activation of asparaginyl endopeptidase (AEP) as a result of brain tissue acidosis and thus dysregulation of inhibitior 2 of protein phosphatase 2 A^53^. Intriguingly, we found by Western blots that the level of tau hyperphosphorylation appeared to recover to normal level at 7 months after CCI, which is in line with dynamic regulation of tau phosphorylation and findings from another group demonstrating that repetitive mild TBI induced only transient increase in phosphorylated tau^54^. In contrast, AT8 immunohistochemical reactivity, which can detect neuronal tau accumulation before the actual formation of neurofibrillary tangles and neuropil threads^42^, persisted in the hippocampus and cerebral cortex 6 weeks to 7 months after CCI, suggesting that CCI induced sustained phospho-tau accumulation in neurons in adult Tg/htau mice. However, the pattern of tau accumulation we observed here was restricted in dentate gyrus and damaged cortical area, similar to that observed in CCI in non-transgenic rats^55^ but different from previous studies in which more broad accumulation of phospho-tau was seen in 3xTg-AD mice with CCI^34^ and in aged Tg/htau mice with repetitive mild TBI^36^, possibly due to different lines of transgenic mice or distinct types of TBI employed. In fact, it is known that tau pathology is difficult to be recapitulated in mice expressing wild-type murine tau only^35^. Hyperphosphorylation of the tau protein has been shown to facilitate its release to extracellular space via an unconventional protein secretion pathway^56^, which is in turn taken up by host neurons and leads to the spread of tau pathology^56–58^. The restricted accumulation of hyperphosphorylated tau observed in the present study could therefore serve as tau seeds that may induce widespread tau pathology late in life.

In human brain the tau protein has six isoforms which vary in the number of microtubule-binding repeats and N-terminal inserts−0N3R, 1N3R, 2N3R, 0N4R, 1N4R and 2N4R taus^59,60^. These isoforms are encoded by a single *tau* gene and result from alternative splicing of the exon 10 (3R-tau or 4R-tau) and exons 2 and 3^59,61^. Alternative splicing of tau is developmentally regulated, from predominantly 3 R tau in fetal brain to approximately 1:1 ratio of 3R-tau/4R-tau in adult human brain^62,63^. Alternative splicing of tau exon 10 has been implicated in tau pathogenesis in neurodegenerative tauopathies^43,64–69^. A system biology study has shown that TBI perturbs genome-wide transcriptional activities, including both expression level and alternative splicing, in the rat brain^70^. Recent studies have suggested that alternative splicing of pre-mRNAs encoding amyloid precursor protein, Bcl-x, extra domain A of fibronectin and prosaposin are implicated in rodent brain in fluid percussion, brain ischemia and facial nerve injury^71–75^. However, in the present study we detected no significant change in levels of 3R-tau and 4R-tau in Tg/htau mice at 6 weeks or 7 months after CCI, indicating that CCI may not induce dysregulation of tau exon 10 alternative splicing on a subacute to chronic basis.

We detected a decrease in synapsin-1, a presynaptic marker, in the ipsilateral compared to contralateral hippocampus 6 weeks after CCI. This indicates that CCI may lead to synaptic deficit or altered synaptic plasticity, which may underlie the cognitive impairment observed in these mice. However, the decrease in synapsin-1 level was not evident by 7 months after CCI, possibly due to compensation in synaptic plasticity. Nonetheless, we observed a consistent decrease in the level of NeuN, a neuronal nuclear marker, in the ipsilateral compared to contralateral hippocampus at both 6 weeks and 7 months following CCI, suggesting potential neuronal loss in the ipsilateral hippocampus induced by CCI. In line with this observation, sustained astrogliosis was evidenced by several folds increase in GFAP in the hippocampus ipsilateral to CCI, although astrogliosis can be a direct consequence of brain trauma *per se*^76,77^. We did not detect significant change of synaptophysin, another presynaptic marker, nor in the level of the post-synaptic marker PSD-95, which differed from the decrease in synapsin-1 level. This could possibly be attributed to differential effect on synaptic markers in certain disease conditions^78^; in other words, synapsin-1 may be more sensitive to CCI than other markers examined.

It should be noted that bilateral hippocampi innervate each other and the axons of the contralateral hippocampus could be damaged in CCI, and therefore the contralateral hippocampus may not be able to serve as an optimal control due to retrograde degeneration. However, statistical comparison with matched observation of the ipsilateral and contralateral sides offers an exceptional opportunity to minimize the influence of animal-to-animal variation. Particularly, we also included a group of age-matched sham surgery mice of the same line as an additional control, and none of the parameters we detected showed statistical significance between the left and right hippocampi in sham control animals. This further validates the contralateral hippocampus as an internal control.

In summary, we found marked cognitive impairment and motor strength/coordination deficit, subacute increase in tau hyperphosphorylation without marked change in tau alternative splicing and subacute to chronic accumulation of phospho-tau with astrogliosis in adult Tg/htau mice after CCI. To the best of our knowledge, this is the first study characterizing Alzheimer-like behavioral, biochemical and immunohistochemical alterations induced by CCI using a mouse line that is transgenic for expression of all six isoforms of non-mutated human tau in a murine tau null background, which may putatively represent the change of tau and tau pathology-associated proteins in human brain with TBI.

## Materials and Methods

### Animals

Tg/htau mice [B6.Cg-Mapt < tm1(EGFP)Klt > Tg(MAPT)8cPdav/J mice] were purchased from The Jackson Laboratory. The Tg/htau line was generated by mating two existing lines, 8c^79^ and tau knock-out mice^80^. The animals were housed in a 12-hour light/dark schedule with free access to food and water. Both male and female mice were used in the present study. Animal use was in full compliance with the NIH guidelines and was approved by the Institutional Animal Care and Use Committee at New York State Institute for Basic Research in Developmental Disabilities.

### Controlled cortical impact

CCI was performed in the left parietal cortex of Tg/htau mice at 5–7 months of age (Fig. 1), by using a protocol established previously^34,81,82^. Briefly, mice were anesthetized, their hair was shaved, and the head was postitioned on a digital stereotaxic frame. A skin incision was made sagittally to expose the skull, in which a 5 mm diameter craniotomy was subsequently made unilaterally between coronal and Lambda sutures, with the aid of a motorized mini-drill. The impact was delivered by using a blunt metal probe, which was 3 mm diameter, 15° angled counter-clockwise, zeroed at the dura mater, and centered at the left parietal area with coordinates −2.7 mm ML/+3.0 mm AP to Lambda with Impact One^TM^ stereotaxic impactor (Leica Biosystems, Richmond, IL). The impact conditions were 5.0 m/s velocity, 100 ms dwell time and 1.5 mm depth. The incision was closed and mice were kept warm on a soft heating pad until fully awake. Animals were randomized into two groups−CCI and sham, and the age and gender were counter-balanced between groups. Sham mice received anesthesia and skin incision only, since craniotomy itself likely results in unexpected brain injury in mice^83^.

Behavior tests assessing learning and memory, anxiety and locomotor activity were performed between 3 and 6 weeks post-CCI. Animals were subjected to less stringent behavioral tests first and then more stringent ones so as to minimize both stress in mice and confounding factors in assessment. Alzheimer-like biochemical and histopathological alterations were analyzed 6 weeks (sub-acute) and 7 months (chronic) post-CCI (Fig. 1**)**.

### Elevated plus maze

Elevated plus maze was conducted to measure anxiety induced by open spaces and height^84^ 3 weeks after CCI. The maze consisted of four 30 cm × 5 cm arms connected by a 5 cm × 5 cm common center area. Two opposite-facing arms were open (open arms, OA), whereas the other two opposing arms were enclosed by 20 cm height walls (closed arms, CA). The entire plus maze was elevated on a pedestal to a height of about 80 cm above floor level. During a single 8-min session, a mouse was placed in the center area. The presence of the animal and the time it spent in different zones were detected by Any-maze video tracking system (Version4.5, Stoelting Co., Wood Dale, IL, USA). For each animal, the number of CA entries, OA entries, and amount of time spent in CA and OA were recorded. The percentage of time spent in OA and the entries into OA were recorded and calculated to evaluate anxiety-like behavior of animals. Urine and defecations were removed and the field was cleaned with 70% ethyl alcohol and air dried between tests of individual animals.

### Open field test

The open field test is widely employed to estimate exploratory activity, anxiety and locomotor activity^85,86^. The testing apparatus was a classical open field with a 50 cm × 50 cm square arena with 40 cm high walls. A 20 cm × 20 cm central area was defined by an automated camera-based video tracking system. The mouse was individually subjected to the test for 15 min at 3 weeks after CCI. The time spent and entries in the center of the arena were recorded as an additional measurement of anxiety. After the 15-min test, mice were returned to their home cages. Between tests of individual mice, the areana was cleaned as described above.

### Novel object recognition

Novel object recognition test was performed to measure learning and memory^87^ at 4 weeks after CCI. The test apparatus was the same one as used in the open field test described above. The procedure consisted of three phases: habituation phase, sample phase, and test phase. During habituation phase, each mouse was allowed to explore the field in the absence of objects for 15 min daily for 4 consecutive days so as to be familiarized with the field. The sample and test phases were designated on the fifth day. During the sample phase, two identical objects were placed symmetrically at 10 cm distance from the walls in the arena. The mouse was placed at the mid-point of the wall away from and with its nose opposing to the objects, and allowed to freely explore for 5 min. The time spent to explore individual objects was recorded. The mouse was reintroduced to the arena for 5 min exploration (test phase) after a 2 h delay. During the test phase, one of the two objects used in sample phase was randomly replaced with a distinctly shaped novel object. Between users, the arena and the objects were cleaned with 70% ethyl alcohol, scented with Airwick to remove olfactory cues, and air dried. Object exploration was defined as touching or sniffing the object. The video tracking system was used to collect behavioral performances automatically. Time spent to explore the novel (T_N_) and the familiar (T_F_) objects were recorded. Discrimination index (DI) was calculated using the following formula: DI = sum of T_N_/(sum of T_N_ + sum of T_F_) × 100%.

### Rota-rod test

Rota-rod test was performed to assess motor coordination and motor learning^41^ at 5 weeks after CCI. The mouse was first trained to stand on the stationery rod in a Rota-rod apparatus (Panlab, LE8500, Spain), and then allowed to run on the rotating rod with a steady acceleration from 4 to 40 rpm over 5 min. The latency at which the mouse fell off the rod was automatically recorded. Each mouse was given six trials with 30 min inter-trial intervals.

### Morris water maze

Morris water maze (MWM) was used to evaluate spatial reference memory^88^ at 5 weeks after injury. The test was performed in a circular tank, with a diameter of 180 cm and a height of 60 cm, filled with water (~21 °C) made opaque by adding non-toxic white paint. The maze was designated two principal axes with each line bisecting the maze perpendicular to each other to divide the maze into four equal quadrants. A platform, 13 cm diameter and submerged 1 cm under the water surface, was placed in the center of one of the four imaginary quadrants of the tank and maintained in the same position during all trials. Each mouse was given 90 s to find the platform. If a mouse did not find the platform in 90 s, it was gently guided to the platform. At the end of each trial, the mouse was left on the platform for 20 s. Three such acquisition trials were administered daily for 4 consecutive days. Each mouse performed a total of 12 trials corresponding to a partial training of the spatial reference memory task. A test for memory retention (probe trial) was executed 24 h after the last day of training. For probe trial, each mouse was allowed to swim for 60 s in the tank in the absence of the escape platform. The swim path, swim distance (cm), escape latency (sec), swimming speed (cm/sec), time spent in each quadrant (sec), distance traveled in each quadrant (cm), latency to enter the target zone (sec), and the number of target zone crossings were recorded in an automated tracking system (Smart video tracking system, Panlab; Havard Apparatus).

### Western blot analysis

At 6 w and 7 m after CCI, mice were euthanized with cervical dislocation without anesthesia or perfusion so as not to affect the authentic phosphorylation state of tau; tau is known to be hyperphosphorylated by anesthesia^89,90^ and very rapidly dephosphorylated during post-mortem delay^91^. Mouse brains were promptly dissected and submerged in ice-cold phosphate-buffered saline (PBS), in which bilateral hippocampi were dissociated from other brain regions. The hippocampal tissue was flash-frozen in dry ice and stored in −80 °C until used for Western blots.

The hippocampus was selected for biochemical analysis because it is a well-known brain region actively involved in learning and memory and it exhibits protein expression profiles that may predispose it to early development of tau pathology in AD^92^. Hippocampal tissue was homogenized in 9 volumes of buffer containing 50 mM Tris-HCl, pH 7.4, 150 mM NaCl, 10 mM β-mercaptoethanol, 50 mM NaF, 1 mM Na_3_VO_4_, 2.0 mM EDTA, 1 mM 4-(2-aminoethyl) benzenesulfonyl fluoride hydrochloride (AEBSF), and 10 μg/ml of each of aprotinin, leupeptin and pepstatin. The brain homogenates were mixed with 2-fold concentrated Laemmli buffer and boiled for 5 min, and the protein concentration was measured by using modified Lowry assay. The same amounts of protein from each sample were separated by sodium dodecyl sulfate (SDS)–polyacrylamide gel electrophoresis (PAGE) and electro-blotted onto PVDF membrane. After blocked with 5% fat-free milk, the membrane was incubated with primary antibodies (Table 1) overnight at room temperature in the presence of 0.1% NaN_3_. After washed with three changes of TBST (Tris-HCl, pH 7.4, 150 mM NaCl, 0.05% Tween 20), the membrane was incubated with the corresponding HRP-conjugated secondary antibody for ~2 h at room temperature. After washed with TBST, the blots were visualized by enhanced chemiluminescence (Thermo Scientific, Rockford, IL) and quantified by densitometry using the Multi Gauge V3.0 software (Fuji Film Co., Ltd., Minato, Tokyo, Japan).

**Table 1:** Primary antibodies employed in the present study.

Antibody	Type	Species	Specificity	Site(s) recognized	Source (catalog #)/reference
Anti-pS^199^-tau	Poly-	R	P-tau	pS^199^	Invitrogen (44734 G)
AT8	Mono-	M	P-tau	pS^202^/pT^205^	ThermoFisherScientific (MN1020)
Anti-pT^205^-tau	Poly-	R	P-tau	pT^205^	Invitrogen (44738 G)
Anti-pS^214^-tau	Poly-	R	P-tau	pS^214^	Invitrogen (44742 G)
12E8	Mono-	M	P-tau	pS^262/356^	Dr. D. Schenk
PHF-1	Mono-	M	P-tau	pS^396/404^	Dr. P. Davies
Anti-pS^262^-tau	Poly-	R	P-tau	pS^262^	Invitrogen (44750 G)
Anti-pS^396^-tau	Poly-	R	P-tau	pS^396^	Invitrogen (44752 G)
92E	Poly-	R	Total tau (murine & human)	/	ref.^93^
RD3	Mono-	M	3 R tau	/	Millipore (05–803)
RD4	Mono-	M	4 R tau	/	Millipore (05–804)
Anti-Syn1	Poly-	R	Synapsin-1	/	Stressgen Biotechnologies (VAP-SV060)
Anti-Syp	Mono-	M	Synaptophysin	/	Millipore (MAB5258)
Anti-PSD-95	Mono-	R	PSD-95	/	Cell signaling (D27E11, #3450)
Anti-NeuN	Mono-	M	NeuN	/	Millipore (MAB377)
Anti-GFAP	Poly-	R	GFAP	/	Millipore (AB5804)
Anti-GAPDH	Poly-	R	GAPDH	/	Santa-Cruz (sc-25778)

*Poly*-, polyclonal; *Mono*-, monoclonal; *S*, sheep; *R*, rabbit; *M*, mouse; *P-tau*, phosphorylated tau.

### Immunohistochemical staining

For immunohistochemical studies, mouse brains were fixed in phosphate-buffered 4% paraformaldehyde at room temperature for 48 h with one change of the fixative. Brain samples were then dehydrated for cryoprotection with phosphate-buffered 30% sucrose before free-floating sections were cut.

Free-floating coronal sections, 40 μm in thickness, of the mouse brain were washed with three changes of PBS, subsequently subjected to permeabilization, blocked with normal goat serum, and then incubated with primary antibodies (Table 1) overnight at 4 °C. After washed in PBS, sections were incubated with Alexa Fluor 555-conjugated species-matched secondary antibodies at room temperature for 2 h, washed with PBS and incubated with nuclear stain TO-PRO 3 Iodide (ThermoFisher Scientific) at room temperature for 15 min. After washed twice in PBS, sections were mounted on microscopic slides, and coverslipped with anti-fade mounting medium. Photomicrographs of the dentate gyrus subfield and the overlaying cerebral cortex, at ~1 mm away from the edge of the cortical tissue loss resulting from impact, were taken with a Nikon EZ-C1 laser scanning confocal microscope. Omission of the primary antibody was employed to serve as a negative control of staining. Since the experimenter were difficult to be kept blind to groups due to significant tissue loss in the cortex after CCI in microscopic assessment, an additional experimenter independently reviewed the stained slides so that potential bias could be minimized.

### Statistical analysis

Data were analyzed with unpaired Student *t* test, matched observation two-way ANOVA or repeated measures ANOVA followed by Bonferroni’s *post hoc* test, where appropriate. The data were expressed as mean ± S.D. *P* < 0.05 was considered statistically significant.
